# The impact of the COVID-19 pandemic on the antimicrobial stewardship workforce in Scottish acute care hospitals—a qualitative study

**DOI:** 10.1093/jacamr/dlae199

**Published:** 2024-12-12

**Authors:** Ayodeji Matuluko, Valerie Ness, Jennifer Macdonald, Jacqueline Sneddon, Ronald Andrew Seaton, Kay Currie

**Affiliations:** Research Centre for Health, Glasgow Caledonian University, Cowcaddens Road, Glasgow G4 0BA, UK; Research Centre for Health, Glasgow Caledonian University, Cowcaddens Road, Glasgow G4 0BA, UK; Research Centre for Health, Glasgow Caledonian University, Cowcaddens Road, Glasgow G4 0BA, UK; Scottish Antimicrobial Prescribing Group, Healthcare Improvement Scotland, Glasgow, UK; British Society for Antimicrobial Chemotherapy, Birmingham, UK; Scottish Antimicrobial Prescribing Group, Healthcare Improvement Scotland, Glasgow, UK; British Society for Antimicrobial Chemotherapy, Birmingham, UK; Queen Elizabeth University Hospital, NHS Greater Glasgow and Clyde, Glasgow, UK; Research Centre for Health, Glasgow Caledonian University, Cowcaddens Road, Glasgow G4 0BA, UK

## Abstract

**Background:**

Antimicrobial stewardship (AMS) programmes seek to reduce the risk of antimicrobial resistance by minimizing inappropriate antimicrobial use. The SARS-CoV-2 coronavirus (COVID-19) pandemic was characterized by initial widespread use of antimicrobials in patients with COVID-19, with potential negative effects on AMS efforts.

**Objective:**

To explore the impact of the pandemic on the AMS workforce in Scottish acute care hospitals.

**Method:**

Individual, semi-structured online interviews were conducted with a purposive sample of clinical staff who had an AMS focused role in Scottish Health Boards. Interviews explored staff experiences of facilitating AMS during the pandemic. Data were analysed using inductive content analysis.

**Results:**

Thirteen staff from seven of 15 Scotland Health Boards participated. The data revealed negative (including staff redeployment and shortages) and positive effects (including improved working relationships and use of technology) on the AMS workforce. Notably, greater appreciation of the work of the AMS team was a positive outcome.

**Conclusions:**

The robust qualitative methods applied in this original study have generated greater understanding of factors that impeded AMS services in Scotland during the pandemic. These findings may resonate internationally. Adaptation to technology and investment in the workforce are recommended to improve the resilience of AMS services in future crises.

## Introduction

To mitigate antimicrobial resistance (AMR), antimicrobial stewardship (AMS) programmes have been implemented globally, focused on antimicrobial use optimization.^1–3^ While progress on AMS has been shown to vary internationally depending on, for example, regulation of antimicrobial prescribing and the level of AMS resource available, the global health crisis caused by the SARS-CoV-2 (COVID-19) pandemic had significant effects on stewardship efforts.^4–6^ Although evidence syntheses eventually revealed little bacterial co-infection with COVID-19,^7–10^ initial clinical uncertainty regarding the aetiology and role of bacterial co-infection and lack of COVID-19 treatments and vaccines led to widespread use of antimicrobials. With limited expertise to treat COVID-19, infectious diseases (ID) and AMS experts were redeployed to the care of COVID-19 patients.^11,12^ Thus, AMS activities were reconfigured, potentially compromising existing progress, particularly in acute care hospital settings.^6,12–14^ While existing quantitative evidence,^6,15–18^ showed the pandemic’s impact on AMR rates, AMS initiatives and antimicrobial consumption, there was a gap in understanding of the pandemic’s impact from the perspective of those working in AMS at the time. In Scotland, a national AMS programme with a collaborative health board approach is coordinated by the Scottish Antimicrobial Prescribing Group (SAPG).^19^ The aim of this paper is to present key aspects of a qualitative study,^20^ which explored the impact of the pandemic on the Scottish AMS workforce.

## Methods

### Ethical approval and access

Glasgow Caledonian University (GCU)’s School of Health and Life Sciences Research Ethics Committee granted ethical approval to conduct this study: reference HLS/NCH/20/011. Formal Management Access Permission was provided by each participating Health Board. It was ensured that interview participants were fully informed and knew that their participation was voluntary (by provision of a Participant Information Sheet). On the interview day, participants were asked to verbally confirm their understanding and agreement to participate in the study, which was recorded and formed the consent to participate.

### Study design

A qualitative study employing semi-structured, one-to-one, online interviews was conducted between March and August 2021 and reported using the consolidated criteria for reporting qualitative research (COREQ),^21^ in Supplementary File 1 (available as Supplementary data at *JAC-AMR* Online).

### Participant recruitment

A purposive sampling strategy was employed. All clinical staff with an AMS focused role [including all leads of the antimicrobial management team (AMT), AMS Pharmacists, and AMS nurses] and associated clinicians working in the ID specialty, e.g. training grade doctors, in the 15 Scottish Health Boards were eligible to participate. Eligible participants were invited to participate via a recruitment email from a gatekeeper in the SAPG network and via advertisement on X^®^. Participants could decide to contact the researcher directly if they wanted to participate, without any form of coercion. Those who responded were provided with the Participant Information Sheet to ensure informed consent.

### Data collection

Interviews were conducted with a topic guide (Supplementary File 2). The guide was developed by A.M., in consultation with V.N., J.M. and K.C. and validated with J.S. and R.A.S. to ensure that sufficient contextual questions were captured. Interview participants were asked to reflect on the period beginning from March 2020 (the onset of the COVID-19 pandemic) until the period of data collection. Open-ended questions were asked about participants’ experiences of facilitating AMS work during the pandemic, including challenges and unexpected benefits; follow-up questions based on participants’ responses were also asked. Online interviews (using Microsoft (MS) Teams^®^) were conducted by A.M. (a female PhD researcher with a MSc in Clinical Pharmacy, a Postgraduate Certificate in research methods and research interests in AMS) and audio-recorded on an encrypted digital recorder. Interviews lasted an average of 36 minutes (ranging from 18 to 58 minutes).

### Data analysis

The data was transcribed verbatim by A.M., managed in NVivo^®^ 12, and analysed via inductive content analysis.^21^ The inductive content analysis followed the steps described by Vears and Gilliam^22^ (familiarization, generation of open codes from line-by-line analysis and grouping similar open codes into subcategories and categories). Generated categories and subcategories were revised several times and an audit trail maintained in NVivo^®^. The analysis was reviewed by V.N. and K.C. to enhance rigour.

## Results

Thirteen staff from seven Scottish Health Boards participated. Participants were in the same role they were in pre-COVID, at the point of data collection. They were three ID consultants (with all being AMT leads), one training grade doctor (rotating within the ID specialty), six antimicrobial pharmacists and three antimicrobial nurses.

Three main categories were generated from the data, with additional subcategories highlighted in Table 1 and within the text, with illustrative participant quotes.

**Table 1.** Categories and subcategories on the impact of COVID-19 on the AMS workforce

Category	Sub-category (where applicable)
1. Negative workforce impact	Change in clinical role Change in working patterns AMS staff shortage
2. Positive workforce impact	Heightened profile of the AMS team Improved working relationships (with the wider MDT and specialties) Use of technology and increased collaboration
3. Influences on capacity to maintain workforce	

### Negative workforce impact

#### Change in clinical role

Changes in the clinical roles of AMS team members and in the team structure resulted from sudden redeployment of staff from their usual AMS duties to general clinical services.

‘One of the biggest things was that our clinical role changed overnight. We went from providing a clinical role to our ID ward to providing a clinical role to the COVID assessment unit.’ Participant 4

‘We have identified some trainees who were interested in helping us to review the effectiveness of prompts [AMS quality improvement (QI) project]. But with COVID these people were redeployed, they went to other areas. So the team infrastructure within the clinical areas fundamentally changed and the purpose of clinical areas changed.’ Participant 5

#### Change in working patterns

Changes in working patterns included changes to working time, more flexible working, staff needing to go on emergency rotas and completely changing their days of working. There was also a limited ability to travel between different sites in particular Health Boards as this was deemed non-essential and a risk for infection transmission. Previously, AMS staff migrated from site to site to cover wards, to review patients and to deliver induction training for new staff. During the pandemic, staff ‘weren't physically able to go and visit some wards to carry out their duties’ (Participant 4).

As participants highlighted, there had been a shift from face-to-face to virtual working, in both reviewing patients and providing AMS education to staff. In some situations, ward rounds became virtual, and the use of the Hospital Electronic Prescribing and Medicines Administration (HEPMA) system increased.

‘We review complex patients with infection, and would normally go to the wards pre-COVID and throughout that 12 months, I was able to cover the two acute sites that had the electronic prescribing quite easily and remotely, so that I was able to contribute to *Staphylococcus aureus* bacteraemia or *C. difficile* reviews.’ Participant 2

#### AMS staff shortage

The impact of AMS staff shortage was a common feature across most of the Boards, influenced in part by the redeployment of staff to cover other clinical areas, as well as AMS team sickness or isolation due to COVID-19. This meant that staff allocation to AMS work was depleted and led to fatigue as reported by a participant.

‘From a ward perspective, there’s just this fatigue within wards to take anything forward as a result of COVID, so it’s just trying to identify the right time, where people are starting to sort of rebuild themselves and rebuild their teams. And because there’s been a lot of reconfiguration with wards, it’s not a normal team…so there’s lots of different barriers to taking forward QI stuff.’ Participant 9

Additionally, AMT leadership was largely disbanded, due to both redeployment and the need to prioritize COVID-related clinical activity. This loss of AMT leadership meant no AMT meetings took place in most sites and there was no leadership to provide direction for AMS efforts.

‘…our stewardship lead was pulled to be COVID lead. So, the stewardship element of their role was completely removed for about a year, and therefore there was nothing coming down from that kind of clinical lead down to our team, for us to action because everything was just stopped.’ Participant 4

‘…most of the people involved in the AMT are also crucial to the COVID response, because we are ID, microbiology, infection control, everything, then it’s very difficult to keep a core activity going.’ Participant 13

Decisions to limit staff presence on wards to only staff essential to the core COVID-19 service, were also influenced by considerations of infection transmission.

‘Another issue at the very beginning why staff were pulled back, was the whole PPE issue. We didn’t really have clear guidance at the beginning. And anxiety I think around that and I suppose the interpretation of, what is essential and what is not; a lot of discussion around that…so again, these are all the things in the early days I think definitely did disrupt our feasibility and ability to be on the wards the way we would be normally.’ Participant 11

### Positive workforce impact

#### Heightened profile of the AMS team

A greater recognition of the AMS team was an unexpected positive outcome of the pandemic highlighted. Previously, AMS teams would have had to introduce themselves to the different medical teams and explain their role. However, during the pandemic staff reported that medical teams were seeking out the AMS teams to ask for their advice.

‘There’s certainly been a lot more recognition of our team following the pandemic, I think. Our team have been quite upfront and central in a lot of the COVID work locally, so we have raised our profile within the organisation. From a stewardship perspective that has certainly been a positive…there’s been a much better appetite for AMS as a result of COVID.’ Participant 9

‘It’s made the hospital more aware of the antimicrobial team. There’s more respect for the antimicrobial team, because we have been involved and we've been there for advice. They were all in a scary place, and anybody that was giving them support earned respect, and I think there’s a respect for the antimicrobial team…they pick up a phone now and ask me advice, whereas before it would be me approaching them.’ Participant 10

This heightened profile and recognition of the AMS team is expected to lead to more progress with implementing future AMS interventions, as one would already be ‘pushing on open doors’ (Participant 7). This may mean that the AMS team will find it easier to collaborate with other specialties and teams in the future.

#### Improved working relationships (with the wider multidisciplinary team and specialties)

As a result of the improved profile of AMS teams—and the aforementioned redeployment of AMS staff to other clinical areas—participants reported improved working relationships with the wider multidisciplinary team (MDT) and clinical specialties they had not engaged with previously. Additionally, the transfer of skills from AMS work to clinical work, and vice versa was also a positive outcome of working in new clinical areas.

‘Some of the positives are that we probably worked very closely as a team, so it probably brought our team a little bit closer together. It allowed us to work with medics and consultants and wider teams that we wouldn’t usually work so closely with, because some of the wider consultants were brought down to our ward to cover shifts within the COVID unit. It allowed us to form relationships and things.’ Participant 4

‘I haven’t worked in a ward in probably over 13 years. So to suddenly be deployed back into a ward environment and had never done a ward round or a drug round in 13 years, I had to upskill pretty quick, but it was good, from an AMS perspective that it made me see how difficult and how challenging ward rounds and drug rounds can be.’ Participant 12

#### Use of technology and increased collaboration

Participants mentioned that the pandemic expedited the implementation of electronic tools. Virtual working was aided by the use of technology. Particularly, the use of software for virtual communication and meetings (MS Teams^®^), and virtual ward rounds enabled wider reach to teams, increased collaborations and wider coverage of more clinical areas. Technological tools are now expected to become a routine aspect of AMS staff’s day-to-day work.

‘A lot of our other sites received virtual microbiology rounds. So the microbiology team would phone in [on Teams] to lunchtime ward review sessions or lunchtime peer review/MDT meeting and have input in that way.’ Participant 4

‘So obviously nobody was working on Teams before. So now we have all got used to working on Teams and there is no doubt that that’s for more effectively communicating than we had ever imagined it would be. And so, you will be able to reach larger audiences more easily, and probably with less organization going forward.’ Participant 5

Technology also influenced the change in the format of the ‘protected policy’ for antibiotics, from paper to electronic. The protected policy process involves a prescriber being required to complete an antibiotic request form before they can prescribe antimicrobials that have been identified as a protected or restricted group of antimicrobials. Pre-pandemic, this was a mostly paper-based process, but it moved swiftly to an electronic process (via HEPMA) with the onset of the pandemic to limit the spread of infection.

‘We suspended our paper-based process, our authorisation process, because we were mindful that paper could become a vector of transmission if a prescriber’s handling it and is reviewing perhaps a COVID infected patient.’ Participant 2

‘Though we had no reasons to believe prescribers were circumventing it and we felt we had a reasonable level of assurance from it, but at the start of COVID because everything went into essential business only, we took down the alert antibiotic system and said “prescribers, you don’t need to do this, but on HEPMA can you please indicate why you’re using an alert antibiotic”.’ Participant 5

However, there could be issues with the use of an electronic version of the protected policy. These issues include inability of some junior staff to access electronic systems and the number of steps involved in the operationalization of the electronic policy, as expanded on by a participant.

‘The paper form made visibility a lot easier. That became a bit trickier, because if you’re doing it electronically, you need to have the electronic access…you can’t really almost automatically give electronic approval to all these people [band 2, band 3 support staff].’ Participant 11

‘I can see how the old system worked and I can see how easy it was to remind doctors, “this is a protected agent, here’s your forms in this drawer, can you fill that out” and you could pass it on to them really easily. Whereas with the electronic form you sort of go up and go, “oh did you know that’s a protected [antibiotic]; you need to go onto [system], link in to this, open the form, electronically fill it in, save it, email it and send”.’ Participant 11

The mode of delivery of AMS education, which used to be via face-to-face induction sessions, also changed. Instead, recorded videos that could be reused and shared across sites were developed, thereby addressing the issues with not being able to travel within sites.

‘Some of our education we re-engineered, so rather than delivering it face-to-face as we used to do. We’ve pre-recorded particularly our induction sessions for new staff throughout that 12-month period. So, we were doing it virtually, sometimes, through Teams, but most often I think a lot of the education from an AMS point of view was delivered remotely through pre-recorded sessions.’ Participant 2

### Influences on capacity to maintain workforce

Having a strong existing AMS team structure in place was pivotal in some sites’ ability to maintain their AMS service. Additionally, empowering other clinical staff with tools and skills needed to do the work themselves, similar to a ‘train-the-trainer’ format, created a sense of ownership (seen as a key factor in maintaining the AMS service).

‘We’ve got a really small team, but we’ve got a big remit, and it’s about ownership at local level. So whenever you're prescribing antibiotics, the best thing we can do is try and help you get it right first time, and then have broad prompts in your area, and perhaps champions to help you deliver better antimicrobial care. We’ve mentored a lot of them through some of the stewardship resources, some of the audits and projects, so a lot of them are running the show now themselves.’ Participant 2

## Discussion

This study was conducted during the second year of the COVID-19 pandemic in the UK (March–August 2021), enabling participants to reflect on their experiences during the first few tumultuous months of the crisis and the impact this had on their work as AMS specialists. At the time of recruitment, while largely quantitative evidence^6,15–18^ was emerging regarding antibiotic use and AMS, there was little published research on the impact of the pandemic from the perspective of those working in the field. Therefore, this qualitative study makes a valuable contribution to the understanding of the causes and consequences of the disruption to AMS services caused by the pandemic.

The study findings revealed negative impacts on the AMS workforce through changes in the clinical role of AMS teams (due to redeployment), changes in working patterns and staff shortages. This provides a qualitative parallel to the work of Wimmer *et al.*,^23^ who reported in a survey of 122 respondents from 68 hospitals in the USA that clinical pharmacists had less time for AMS, with 75% reported as having redeployed to COVID-19 related leadership positions. Similarly, a UK survey^18^ with AMS leads (*n* = 95; out of 169 NHS Trusts or Health Boards) found that 57% of antimicrobial pharmacists were seconded to other roles within the wider clinical and pharmacy team, corroborating findings from our study. More recently, a qualitative study^24^ with 17 members of AMS teams from 17 hospitals in the UK found that AMS staff redeployment in the midst of conflicting COVID-19 priorities negatively affected the continuation of AMS activities. The findings from our Scottish study, corroborate these findings from across the UK with additional unique insights from AMS nurses (although few in number in Scotland^19^), who were less represented in the former UK studies.^18,24^ Changes in working patterns that resulted in more flexible working, including carrying out activities (such as AMS rounds and education) remotely, have remained following the pandemic, as highlighted by SAPG.^19^ Furthermore, while the changes in clinical roles of AMS team members—brought about by the pandemic—were temporary, these changes facilitated wider reach to other clinical areas, as our participants discussed.

Clearly, international evidence shows that redeployment of staff, while operationally necessary at the time, had a significant impact on continuation of AMS services. Greater investment into the AMS workforce is pertinent, with training of all AMS healthcare professionals (HCPs) needed. The antimicrobial nurses (*n* = 3) who were participants in this study commented on the value that they believe they bring to AMS teams, and their perception that there is insufficient recognition of AMS nurses at the hospital management level. Thus, additional investments into the specialist antimicrobial nursing workforce in Scotland would be beneficial, to address the current limited antimicrobial nursing posts, as also reported in the SAPG workforce report.^19^ Furthermore, based on the workforce gaps identified in our study, empowering ward nurses would be one mechanism to strengthen AMS activity more broadly and potentially counter the impact of redeployment of specialists in future pandemics. Chater *et al.*^25^ have shown that empowering and training nurses has a positive impact on AMS behaviours. Additionally, Fisher *et al.*^26^ found that barriers to nurses’ promotion of the principles of intravenous to oral antibiotic switch therapy, included lack of knowledge, confidence and poor relationships with prescribers. Thus, better engagement and training of all ward nurses, and their recruitment to AMS teams should help strengthen AMS programmes, particularly during pandemics.

Positive effects on the AMS workforce that were reported in this study include a heightened profile of AMS (i.e. through more acknowledgement of the need for AMS and a greater appreciation of the skills of the AMS team), improved working relationships with the wider MDT and the increased use of technology (for AMS education, antibiotic review and virtual ward rounds) fostering collaboration. These findings exist against a backdrop of national AMS interventions by SAPG during the pandemic, in the form of reissued guidance on antimicrobial prescribing and the deployment of a national point prevalence survey in patients hospitalized with suspected COVID-19.^16,27^ The latter intervention was potentially important in raising the profile of AMS as it involved engagement with AMS teams throughout Scotland including many training grade doctors, with the key elements of AMS being maintained.^16^

Some of our findings are similar to recent studies conducted within and outside the UK. In the United Arab Emirates, Hashad *et al.*^28^ in a qualitative study with 31 participants across 11 hospitals highlighted the increasing complexity of AMS implementation and how improved collaborations (aided by the use of technology, to facilitate AMS team meetings and virtual rounds) within the MDT were key to maintaining AMS during the pandemic. Findings from our study revealed that participants considered the increased use of technology for virtual education a positive consequence of the pandemic. This is corroborated by a survey by Chauhan *et al.*^29^ who invited HCPs working in AMS in Wales to provide data on educational sessions delivered in their facilities, including numbers and lengths of sessions, and teaching format. In their study,^29^ although the ability to provide AMS education in the previous face-to-face formats was impaired due to COVID-19, the use of virtual education allowed facilities to reach an increased number of staff while providing shorter and fewer sessions. This use of virtual education formats seems to be a positive feature of the pandemic and should continue into the future to strengthen the provision and reach of AMS education.

### Strengths and limitations

This qualitative study provides unique insights into the perspectives of AMS team members on the impact of the pandemic on their AMS activity in Scotland. As such, it adds to the knowledge base of the causes and consequences of disruptions to AMS services during COVID-19, pointing to both negative and positive outcomes.

This study has included a sample size of 13 participants (from 7 out of 15 Scottish Health Boards), which Francis *et al.*^30^ has proposed as sufficient for a qualitative study. Recruitment constraints brought about by the COVID-19 pandemic hampered the ability to recruit more participants from other Scottish Health Boards. Thus, while there may be limitations in the transferability of the findings generated to the other eight Boards (considering the existing AMS teams’ structures (pre-COVID), differences in patient load, clinical specialties and geographical location), it is deemed that sufficient data were provided from a broad range of Boards (small to medium-sized to large populations served, within rural and urban locations) to address the research objective, evidenced by the similarities in the responses of interviewees, with little new data gathered from later interviews.

### Implications for practice

A key practical benefit of changes to working practices during the pandemic was the increased use of technology for both patient review (via HEPMA, MS Teams consultations and telephone calls) and online staff education (through remote participation in training and easier access to recorded training sessions). This benefit should be capitalized on in the adaptation of the mode of delivery of AMS education initiatives and the utility of electronic prescribing tools, in future. However, care should be taken to ensure that the use of electronic systems for aspects such as the protected policy for antibiotics, do not introduce barriers to ensuring AMS.

Furthermore, additional investments into the AMS workforce (particularly nurses) will be pivotal in ensuring there is an adequate level of human resource to maintain AMS services during periods of displacement. Additionally, capitalizing on the improved profile of AMS and better working relationships with the wider MDT—as a result of the COVID-19 pandemic—is important to sustain progress with AMS.

### Conclusion

The robust qualitative methods applied in this original study have generated a greater understanding of the factors that impeded the delivery of AMS services during the pandemic, particularly AMS team redeployment. However, unanticipated positive outcomes such as raising awareness of the value of AMS teams and greater use of technology in patient management and staff communication also have merit. These findings have significance in highlighting opportunities to act to improve the resilience of AMS services in the UK and globally, during future crises.

## Supplementary Material

dlae199_Supplementary_Data
