# Supplementary material for: The impact of the COVID-19 pandemic on the antimicrobial stewardship workforce in Scottish acute care hospitals—a qualitative study
Source: JAC Antimicrob Resist. 2024 Dec 12;6(6):dlae199. doi: 10.1093/jacamr/dlae199 (PMC11635100; doi:10.1093/jacamr/dlae199)
Supplement: dlae199_Supplementary_Data [file dlae199_supplementary_data.zip › Supplementary file 2_Interview Topic Guide_Impact of the COVID-19 pandemic on the AMS workforce in Scotland.docx]

**Supplementary file 2**

**Interview Topic Guide** *(intended to be used flexibly, with follow-up questions based on participants’ responses)*

1. Firstly, can you tell me how Antimicrobial Stewardship (AMS) in your area has been impacted due to the COVID pandemic?
2. What has been your experience of facilitating AMS QI work during the COVID pandemic?

Prompt:

- 1. What have been the challenges?
  2. Has anything been easier than expected, or any unexpected benefits of COVID on AMS?

1. What current strategies do you have in place in your area to facilitate early review and documentation of the duration of antibiotics?*

**End of interview**: Thanks for your time. Do you have any questions, further points to highlight? You can also phone or email me after

**Interview question focused on assessing the implementation of a strategy to improve antibiotic review, with follow up questions informing a parallel evaluation, not reported in this current paper.*
